# The single-cell immune atlas of bacterial pneumonia: from inflammatory cell infiltration to treatable cell states

**DOI:** 10.3389/fcimb.2026.1889185

**Published:** 2026-09-10

**Authors:** Hang Chen, Yan Xu, Zhiwei Guan, Jianli Qiu, Xinyu Xu, Suping Yu, Shuhua Fan, Guihua Song, Xia Zhang, Xianqing Ren

**Affiliations:** 1 Department of Pediatrics, The First Affiliated Hospital, Henan University of Chinese Medicine, Zhengzhou, China; 2 School of Pediatrics, Henan University of Chinese Medicine, Zhengzhou, China

**Keywords:** bacterial pneumonia, host-directed therapy, immune atlas, immunosuppressive macrophages, neutrophil heterogeneity, single-cell RNA sequencing, T-cell exhaustion, treatable cell states

## Abstract

Bacterial pneumonia remains a major cause of infectious mortality, and antimicrobial resistance has intensified interest in host-directed therapy (HDT). Recent bronchoalveolar lavage fluid (BALF) and peripheral blood atlases (2024–2025) provide single-cell evidence from human disease, but the main translational gap is prioritization rather than catalog expansion. In this review, we reorganize published datasets from bacterial pneumonia and related lung infection cohorts (>500,000 cells; 2020–2026) into a state-prioritization scheme termed “treatable cell states.” Evidence is currently strongest for severity-linked expansion of indoleamine 2,3-dioxygenase 1–positive/programmed death-ligand 1–positive (IDO1+/PD-L1+) macrophages and pathological neutrophils; exhausted-like CD8+ T-cell states show emerging human transcriptomic and translational support but still require protein-level, functional, and longitudinal validation, whereas evidence for injured epithelium and repair-failed stromal states in bacterial pneumonia remains partly inferential and draws on adjacent lung-injury literature. We therefore treat the six proposed state categories as a ranked hypothesis set for macrophage-, neutrophil-, T-cell-, epithelial-, and stromal-directed interventions, not as validated clinical targets. Most proposed interventions still require longitudinal and functional validation before routine bedside use.

## 1 Introduction

Lower respiratory tract infections remain a leading cause of infectious mortality worldwide. The Global Burden of Disease (GBD) 2019 study estimated more than 2.5 million deaths annually, with *Streptococcus pneumoniae*, *Klebsiella pneumoniae*, and *Staphylococcus aureus* as major pathogens in community-acquired and hospital-acquired pneumonia (Torres et al., 2021; GBD 2019 LRI Collaborators, 2022). Despite vaccines and antibiotics, multidrug resistance, sepsis-associated immune paralysis, and high mortality in severe disease continue to limit antimicrobial-only treatment strategies (Mizgerd, 2008; Metlay et al., 2019). These clinical constraints have increased interest in host-directed immune modulation as an adjunct to pathogen-directed therapy.

Progress now depends on resolving host immune states at the site of infection, but prior work relied mainly on peripheral blood and animal models. Blood sampling is accessible but does not fully capture lung-local immune reprogramming, and interspecies differences limit direct translation of animal findings to human disease (Traber and Mizgerd, 2025; Xiao et al., 2025b). Single-cell RNA sequencing (scRNA-seq) helps close this gap by resolving cell states in human samples. In 2024–2025, two large atlases—one based on bronchoalveolar lavage fluid (BALF; 74 patients, 444,146 cells) and the other on peripheral blood (100 patients)—provided the first large-scale single-cell views of bacterial pneumonia (Xiao et al., 2025a; Xiao et al., 2025b). This sets up the central question of this review: not whether additional subsets can be identified, but whether identified subsets can be prioritized for therapeutic testing.

Guided by the “treatable traits” concept in respiratory precision medicine (Xie et al., 2025), we use “treatable cell states” as a prioritization scheme for cell programs that are associated with severity or adverse outcomes and have plausible intervention points. Unlike treatable traits, which are anchored at phenotype/biomarker level, treatable cell states are anchored at cellular programs resolved by single-cell data. This review integrates BALF and peripheral blood evidence to summarize six candidate states: indoleamine 2,3-dioxygenase 1–positive/programmed death-ligand 1–positive (IDO1+/PD-L1+) immunosuppressive macrophages, macrophages with a monocytic myeloid-derived suppressor cell (M-MDSC)-like phenotype, pathological neutrophils, exhausted-like CD8+ T cells, injured alveolar epithelium, and repair-failed stromal cells. Our intent is to rank evidence strength and translational readiness across these states and to identify where host-directed therapy (HDT) proposals remain hypothesis-generating rather than clinically testable.

## 2 Single-cell immune atlas of bacterial pneumonia

### 2.1 Study design and technology platforms

The two most representative single-cell atlas studies in bacterial pneumonia to date focus on BALF and peripheral blood, respectively, providing complementary datasets. The BALF atlas study enrolled 74 patients with bacterial pneumonia and healthy controls, and used the 10x Genomics platform to perform scRNA-seq on 444,146 cells, covering major cell lineages including alveolar macrophages, monocyte-derived macrophages, neutrophils, T cells, B cells, epithelial cells, endothelial cells, and fibroblasts (Xiao et al., 2025a). The peripheral blood atlas study enrolled 100 patients and systematically characterized transcriptomic alterations of circulating immune cells and their associations with pulmonary infection severity (Xiao et al., 2025b). In addition, BALF single-cell studies of pneumonia secondary to sepsis provided complementary evidence on neutrophil dysfunction in severe and immunosuppressed states (Wang et al., 2021; Shen et al., 2025), and a scRNA-seq study of BALF in pediatric *Mycoplasma pneumoniae* pneumonia provided information on pathogen-specific immune cell states (Shen et al., 2024). Related single-cell studies in viral pneumonia also provide useful comparative context: BALF profiling in COVID-19 resolved lung-local immune heterogeneity (Liao et al., 2020), while severe COVID-19 was characterized by marked dysregulation of the myeloid compartment (Schulte-Schrepping et al., 2020). In addition, single-cell immune repertoire analysis provides a complementary methodological framework for characterizing adaptive immune heterogeneity (Irac et al., 2024). Together, these studies have generated a dataset spanning local (BALF) and systemic (peripheral blood) compartments, adult and pediatric populations, and community-acquired and sepsis-associated bacterial pneumonia.

All of the above studies were based on the 10x Genomics platform, and the related technical biases impose certain limitations on data interpretation. Fragile cells such as neutrophils have lower viability in conventional droplet-based capture workflows, which may systematically underestimate the actual proportions of neutrophil subsets; the dropout effect for low-abundance transcripts decreases the detection sensitivity of marker genes and introduces uncertainty in the identification of rare subsets; in addition, the influence of doublet rate on the identification of rare cell states must be corrected during analysis (Gulati et al., 2025). Therefore, the cell subset proportions and gene expression levels reported in current atlases are more appropriate for qualitative comparison of transcriptionally defined cell programs than for precise quantitative measurement of functional cell states, and quantitative or functional conclusions await further validation using improved cell-capture and protein-detection workflows, such as cellular indexing of transcriptomes and epitopes by sequencing (CITE-seq) or related multimodal approaches.

Throughout this review, we explicitly distinguish evidence derived from human BALF, human peripheral blood, pediatric BALF, sepsis-associated BALF, animal models, ex vivo functional assays, and adjacent lung-injury contexts. Human BALF data are treated as the most direct evidence for lung-local cell states in bacterial pneumonia. Peripheral blood data are considered complementary systemic evidence, but not a direct substitute for lung-local states unless paired BALF–blood validation is available. Animal and adjacent-disease data are used to support mechanism or intervention plausibility, but not to establish clinical readiness in bacterial pneumonia.

### 2.2 Cellular composition and its association with disease severity

**Figure 1:**
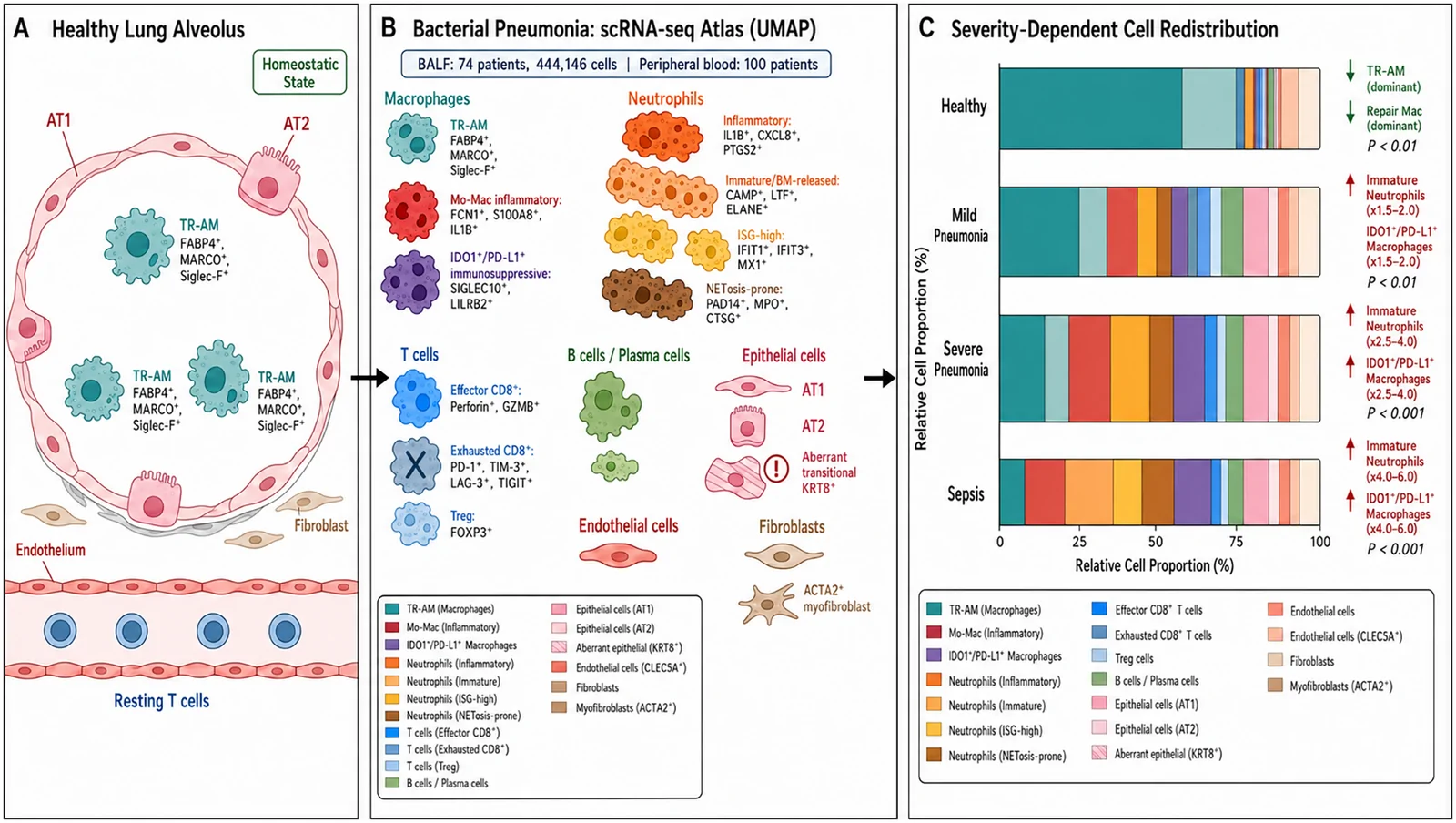
Single-cell atlas overview of bacterial pneumonia. **(A)** shows homeostatic alveoli; **(B)** shows BALF UMAP clusters across immune and structural lineages; **(C)** summarizes severity-associated redistribution of major BALF immune-cell states based on published bacterial pneumonia BALF atlas data and related sepsis-associated BALF studies. The healthy condition is shown as a qualitative macrophage-dominant reference rather than as a pooled quantitative comparator. Because the contributing datasets differ in cohort design, sampling compartment, disease context, and cell-capture efficiency, this panel should be interpreted as a schematic summary of directional changes rather than as a formal meta-analysis of absolute cell proportions.

The cellular composition of infected lung tissue in bacterial pneumonia differs markedly from that of healthy alveoli. Under physiological conditions, alveolar macrophages (AMs) are the principal immune cell population in the alveolar space and are responsible for immune surveillance and the maintenance of surfactant homeostasis (Hussell and Bell, 2014; Traber and Mizgerd, 2025). After bacterial infection, large numbers of monocyte-derived macrophages, neutrophils, and T cells infiltrate the infection site from the circulation, and the cellular composition of BALF undergoes substantial changes (Xiao et al., 2025a). These changes are reflected not only in differences in total cell numbers but, more importantly, in shifts in the proportions of subsets with specific transcriptional programs or inferred functional orientations. In severely affected patients, the proportions of immature and interferon-stimulated gene (ISG)–high neutrophils in BALF are significantly elevated (approximately 2.5–4.0 times those in mildly affected patients, *P* < 0.01), and the proportion of IDO1^+^/PD-L1^+^ immunosuppressive macrophages is approximately 3.2 times that in mildly affected patients (P < 0.001), while macrophage subsets with tissue repair functions are correspondingly reduced (Xiao et al., 2025a; Lin et al., 2026). These differences suggest that pneumonia severity is associated with an imbalance among transcriptionally defined immune-cell programs, including immunosuppression-associated macrophage programs, pathological neutrophil programs, and reduced repair-associated macrophage signatures. Thus, severe pneumonia appears to be characterized not only by increased inflammatory-cell infiltration, but also by severity-associated redistribution of specific BALF cell programs (**Figure 1**).

### 2.3 Complementary perspectives from BALF and peripheral blood atlases

The interplay between local pulmonary immunity and systemic immune responses is an important dimension for understanding the immunopathology of bacterial pneumonia. Peripheral blood scRNA-seq data show that patients with bacterial pneumonia exhibit substantial expansion of myeloid cells and an exhausted-like phenotype in T cells in the circulation, and that these peripheral changes are positively correlated with pulmonary infection severity (Xiao et al., 2025b). In peripheral blood, *S100A8/A9*-high monocytes drive systemic inflammation through the Toll-like receptor 4 (TLR4)–myeloid differentiation primary response 88 (MYD88) signaling axis, and the expansion of M-MDSC–like cells is closely associated with T cell suppression (Sanchez and Kulkarni, 2025; Xiao et al., 2025b). This pattern of pulmonary infection driving peripheral immune reprogramming indicates that the impact of localized infection on the systemic immune system is broader than previously recognized; integrated analysis of BALF and peripheral blood atlas data can more comprehensively depict the immunopathological process from the infection site to the circulation and support development of peripheral blood–based monitoring and prognostic tools (Sanchez and Kulkarni, 2025).

Current BALF and peripheral blood atlases are largely independent datasets, and the main unresolved issue is the lack of truly paired BALF–blood samples from the same patients. Cross-platform methods, including Harmony, canonical correlation analysis implemented in Seurat (Seurat CCA), and single-cell variational inference (scVI), facilitate dataset alignment (Lyu et al., 2025), but without paired sampling they cannot determine whether blood signatures can reliably proxy lung-local states. The immediate priority is therefore paired longitudinal cohort design for direct cross-tissue state tracking.

## 3 Macrophages: transition from tissue residence to immunosuppression

### 3.1 Coordinated remodeling of alveolar and monocyte-derived macrophages

Macrophages are central effector and regulatory cells of pulmonary innate immunity. Under physiological conditions, tissue-resident alveolar macrophages (TR-AMs) maintain surfactant homeostasis and low-level surveillance (Hussell and Bell, 2014). Their maintenance relies on GM-CSF/PPAR-γ signaling and expression of homeostatic markers such as *FABP4* and *MARCO*. After bacterial infection, TR-AMs rapidly shift toward inflammatory programs (e.g., *TNF*, *IL1B*, *CXCL2*) while downregulating homeostatic genes (Xiao et al., 2025a; Lin et al., 2026). With ongoing injury, part of the TR-AM compartment adopts pro-resolution programs, whereas severe inflammation can deplete TR-AMs through inflammatory cell death, creating space for monocyte-derived macrophage recruitment.

Concurrent with these TR-AM changes, circulating Ly6C-high monocytes are mobilized to infected lung tissue and recruited largely through the CCL2–CCR2 axis (Xiao et al., 2025a; Lin et al., 2026). After endothelial transit, they differentiate locally into monocyte-derived macrophages (Mo-Macs). Atlas data separate Mo-Macs into transcriptionally defined proinflammatory (*FCN1/S100A8/IL1B*), immunosuppression-associated (*PD-L1/IDO1/SIGLEC10*), and intermediate programs, with pseudotime supporting a putative transition from inflammatory to suppressive transcriptional programs (Xiao et al., 2025a; Lin et al., 2026). A Ly6G-high macrophage program linked to TREM2 has also been associated with epithelial regeneration after injury (Ruscitti et al., 2024). Overall, these findings support a dynamic macrophage spectrum rather than fixed M1/M2 categories.

### 3.2 Transcriptional identification and functional inference of PD-L1+/IDO1+ macrophages

In the BALF single-cell atlas of bacterial pneumonia, a macrophage subset (Macro_03_M1) co-expressing *IDO1* and *PD-L1* was identified as an independent transcriptional cluster with immunosuppression-associated features (Xiao et al., 2025a). This subset highly expresses indoleamine 2,3-dioxygenase 1 (*IDO1*; log_2_FC > 2.5, FDR < 0.001) and programmed death-ligand 1 (*PD-L1/CD274*), and co-expresses immunosuppressive receptors such as *SIGLEC10* and *LILRB2*. *SIGLEC10*, a member of the sialic acid–recognizing immunoglobulin-like lectin family, can transmit inhibitory signals upon binding CD24 and may reduce macrophage phagocytic activity or proinflammatory cytokine production in appropriate ligand contexts; *LILRB2* can inhibit immune receptor signaling by recruiting SHP-1/SHP-2 phosphatases (Xiao et al., 2025a). Whether these inhibitory receptor programs are functionally active in bacterial pneumonia lesions still requires direct validation. Comparative transcriptomic analysis indicates that this subset shares partial gene expression features with M-MDSCs in the tumor microenvironment (including co-expression of *ARG1*, *NOS2*, and *TGFB1*), but the two differ in regulatory pathways and metabolic features. IDO1-high macrophages in bacterial pneumonia are induced primarily through the interferon-γ (IFN-γ)/STAT1 and Toll-like receptor (TLR)/MyD88/NF-κB signaling axes, whereas counterparts in the tumor microenvironment depend more on transforming growth factor-β (TGF-β)/SMAD and interleukin-10 (IL-10)/STAT3 signaling (Xu et al., 2025). Differences in upstream regulatory signals are reflected in distinct metabolic enzyme profiles: IDO1-high macrophages in the infectious context show transcriptional enrichment of tryptophan-metabolism-related genes, suggesting possible activation of the kynurenine pathway, whereas counterparts in the tumor microenvironment show greater alterations in arginine-metabolism-related programs.

Evidence for this macrophage program is layered rather than uniform. Co-expression of IDO1 and CD274 (PD-L1) is directly observed in BALF single-cell datasets (Xiao et al., 2025a). The link between IDO1-high myeloid states and T-cell suppression is supported by bacterial infection and sepsis models (Xu et al., 2025). By contrast, several downstream steps—especially the full tryptophan–GCN2–AhR cascade and the magnitude of PD-1/SHP-2 signaling effects in bacterial pneumonia lesions—are still inferred in part from non-infection contexts such as tumor immunology. In this review, we interpret these mechanisms as biologically plausible working models: IDO1-mediated tryptophan depletion may limit T-cell translation and proliferation, kynurenine-AhR signaling may favor regulatory programs, and PD-L1/PD-1 signaling may reduce TCR activity; these pathways likely interact, but their causal hierarchy in human bacterial pneumonia remains to be resolved.

Cross-disease comparative analyses help further characterize the biological features of infection-associated immunosuppressive macrophages. IDO1-high and PD-L1-high macrophages in bacterial pneumonia partially overlap with tumor-associated macrophages at the transcriptomic level and share immunosuppressive gene modules including *CXCL10*, *CCL22*, and *TGFB1* (Xiao et al., 2025a; Xu et al., 2025). However, the regulatory mechanisms of this subset in the infectious context have distinct features: IDO1 induction is driven primarily by IFN-γ and TLR signaling rather than by the TGF-β– and IL-10–dominated signaling of the tumor microenvironment. From a therapeutic perspective, the reversibility of infection-associated immunosuppressive macrophages may be greater than that of their tumor counterparts: once the infection is controlled, the inflammatory signals driving their differentiation diminish; in contrast, immunosuppressive signals in the tumor microenvironment are persistent and self-reinforcing (Zhang et al., 2024; Lin et al., 2026). Similar immunosuppressive macrophage subsets have also been reported in scRNA-seq studies of immune checkpoint inhibitor–related pneumonitis and tuberculosis (Zumla et al., 2016; Zhang et al., 2024; Lin et al., 2026). A cautious interpretation is that severe bacterial pneumonia engages a putative and potentially reversible macrophage suppression-associated program that partially overlaps tumor immune-escape modules but remains infection-context dependent.

### 3.3 Limitations of the M1/M2 dichotomy and the concept of a functional continuum

The traditional macrophage polarization model classifies macrophages into two mutually exclusive functional states: classically activated (M1, induced by IFN-γ/lipopolysaccharide [LPS] and characterized by high expression of iNOS, TNF, and IL-12) and alternatively activated (M2, induced by IL-4/IL-13 and characterized by high expression of *Arg1*, *CD206*, and *IL-10*). This classification framework, derived from *in vitro* polarization experiments, has some value for describing basic functional tendencies of macrophages; however, single-cell transcriptomic data have revealed its limitations in the complex inflammatory environment *in vivo*. In bacterial pneumonia BALF, macrophages do not appear as discrete M1 or M2 polarized clusters but are instead distributed along a continuous transcriptional–functional spectrum, with individual cells co-expressing different combinations of traditional M1 markers (e.g., *IL1B*, *CXCL9*) and M2 markers (e.g., *CD163*, *CCL22*) (Xiao et al., 2025a; Lin et al., 2026). This pattern of co-expression is unlikely to be explained solely by technical noise and may reflect the integrated transcriptional response of single macrophages receiving multiple microenvironmental signals simultaneously: bacterial products and IFN-γ drive transcription of proinflammatory genes, whereas local tissue repair signals and phagocytosis of apoptotic cells activate anti-inflammatory and repair-related genes; these two types of signals are not mutually exclusive within the same cell but may coexist with different weights.

Human immunophenotyping studies provide protein-level support for this continuum model. In healthy human pulmonary mucosa, alveolar macrophages often co-express classical M1-associated surface markers, such as CD80, CD86, and CD64, together with M2-associated markers, such as CD206 and CD163, rather than segregating into mutually exclusive M1-like or M2-like populations. In BALF from inflammatory lung disease, macrophages that are not readily classifiable by conventional M1/M2 surface-marker combinations may still display pro-inflammatory transcriptional signatures. These findings provide a useful bridge between single-cell transcriptomic states and surface protein phenotypes, and support the interpretation that human alveolar macrophage activation is multidimensional rather than binary (Mitsi et al., 2018; Takiguchi et al., 2021).

**Figure 2:**
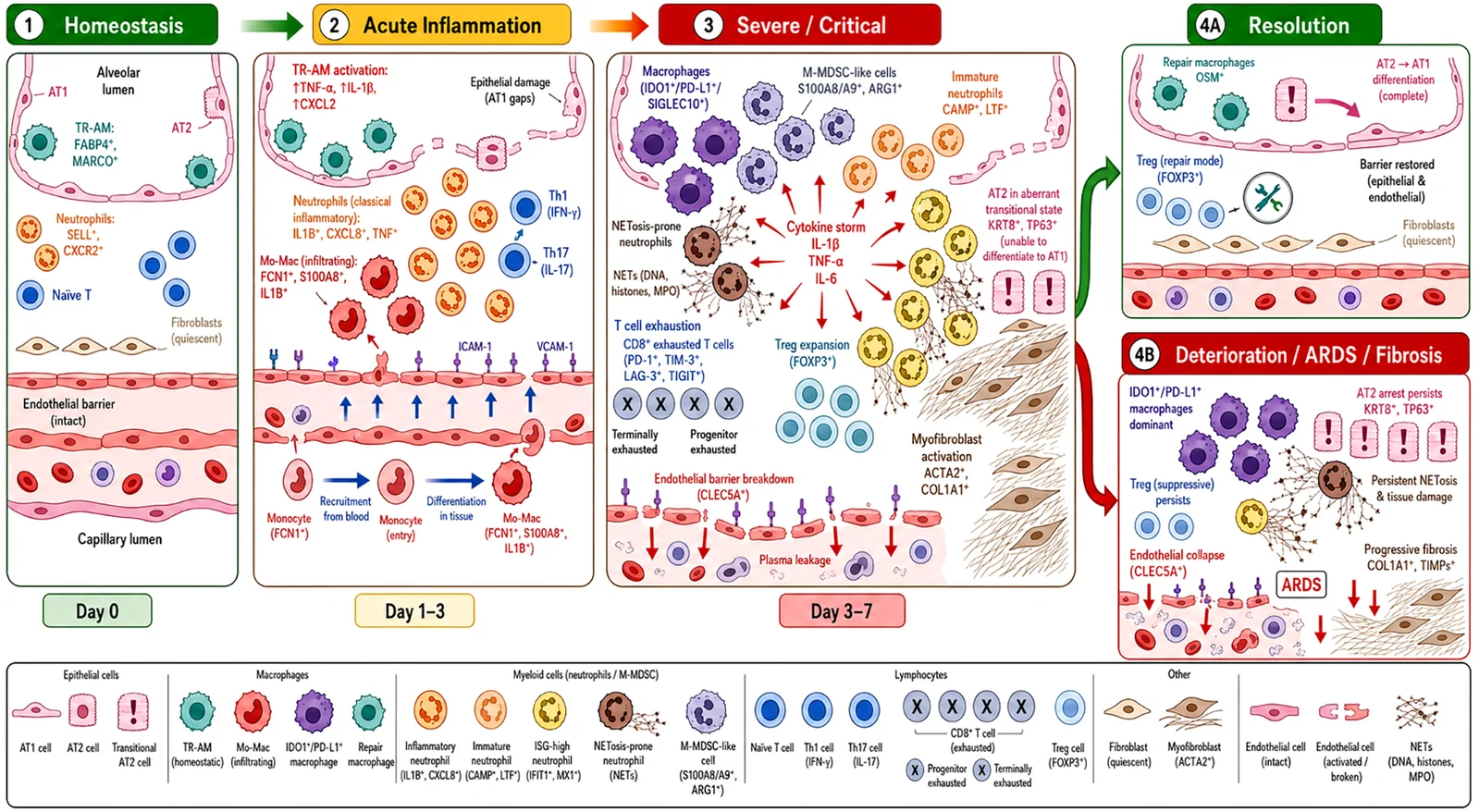
Dynamic state transitions during bacterial pneumonia. The timeline summarizes progression from homeostasis to early infection, severe/critical disease, and bifurcation toward resolution or worsening. It highlights coordinated shifts in macrophage, neutrophil, T-cell, epithelial, endothelial, and fibroblast states rather than fixed M1/M2 or single-lineage transitions.

Pseudotime analysis and RNA velocity results further support the concept of a transcriptional continuum with inferred functional orientations from a kinetic perspective. The transition of macrophages from a proinflammatory transcriptional program through several intermediate programs toward immunosuppression-associated or tissue-repair-associated programs appears to proceed as a continuous transcriptomic evolution rather than as discrete jumps between two fixed extremes (Lin et al., 2026). Diffusion map and partition-based graph abstraction (PAGA) analyses likewise support the continuous distribution of macrophage transcriptional states with inferred functional orientations after dimensionality reduction (Li et al., 2025). The Macro_03_M1 subset is a representative example: although its name follows the traditional M1 label, it exhibits both M1-like features, such as high expression of proinflammatory cytokine receptors, and an immunosuppression-associated transcriptional profile characterized by high IDO1 and PD-L1 expression, and therefore cannot be assigned to either polarization category. The functional continuum model has implications for therapeutic strategy: targeted intervention should be directed at specific positions and regulatory nodes along the functional spectrum of macrophages, rather than broadly inhibiting or promoting M1 or M2 polarization. In severe bacterial pneumonia, a future therapeutic hypothesis is not to fully suppress all M2-like activity, but to determine whether the IDO1-high and PD-L1-high macrophage program can be selectively modulated while preserving phagocytic and tissue-repair-related functions (**Figure 2**).

### 3.4 Macrophage–T cell immune checkpoint interaction network

Within the alveolar microenvironment of severe bacterial pneumonia, macrophages and T cells form a multilayered immune checkpoint interaction network whose complexity exceeds what any single receptor–ligand pair can describe. Single-cell ligand–receptor interaction analyses predict that PD-L1-high macrophages may contribute to suppression of CD8+ T-cell cytotoxic programs in BALF through the PD-1/PD-L1 axis, with reduced expression of cytotoxic molecules such as perforin and granzyme B (Xiao et al., 2025a). However, this inferred interaction requires confirmation by protein-level checkpoint profiling and ex vivo co-culture or blockade experiments. In addition to the PD-1/PD-L1 axis, multiple parallel immune checkpoint pathways contribute synergistically to T cell suppression. Based on evidence from bacterial pneumonia ligand–receptor analysis and related immune contexts, Galectin-9/TIM-3 signaling may contribute to T-cell dysfunction or apoptosis through calcium influx and Bax/Bak-mediated mitochondrial pathways, but whether this pathway is functionally dominant in bacterial pneumonia BALF remains to be validated (Feng et al., 2025; Xiao et al., 2025a). CD80 and CD86 on macrophages may engage CTLA-4 on T cells, compete with CD28 for B7 ligands, and reduce T-cell receptor signaling strength and IL-2 production through inhibitory signaling pathways, although direct functional validation in bacterial pneumonia lesions remains limited (Feng et al., 2025; Xiao et al., 2025a). The synergistic action of multiple checkpoint pathways renders the immunosuppressive effect independent of the activation strength of any single pathway, allowing the suppressive network to maintain considerable T cell inhibition even when individual pathways are perturbed.

In sepsis models, CD47–amyloid-β precursor protein (APP)–CD74 signaling has been shown to trigger adaptive immunosuppression and exacerbate immune paralysis (Feng et al., 2025). CD47 transmits a “don’t-eat-me” signal that inhibits phagocytosis upon binding SIRPα on macrophages, whereas APP–CD74 binding activates p50 homodimers, the inhibitory subunits of NF-κB, downregulates antigen presentation and costimulatory molecule expression on macrophages, and indirectly suppresses T cell reactivation and effector differentiation (Feng et al., 2025). In the infectious microenvironment, activation of this immune checkpoint signaling network has dual biological significance: it helps limit immunopathological damage and preserve tissue integrity during early infection, but in severe infection it may lead to excessive suppression of adaptive immune responses and result in secondary immune paralysis. From a therapeutic standpoint, the multinodal immune checkpoint network in the lung microenvironment supports a mechanistic hypothesis for combinatorial immune restoration: simultaneous modulation of PD-1/PD-L1 and IDO1 may be more effective than targeting either pathway alone, but its efficacy and safety in bacterial pneumonia remain unproven and the risk–benefit balance must be evaluated carefully in the specific pathophysiological context of infection (see Section 8).

## 4 Neutrophils: functional heterogeneity and their bidirectional role

### 4.1 Single-cell–based classification of neutrophil subsets

Neutrophils are the most abundant infiltrating immune cells in bacterial pneumonia and have long been regarded as relatively homogeneous, short-lived effector cells. Single-cell datasets revise this view. Across BALF atlases, neutrophils can be grouped into transcriptionally defined programs annotated as inflammatory (*IL1B/CXCL8*), immature (*CAMP/LTF*), interferon-responsive (*IFIT1/ISG15*), NET-associated (*PADI4/MPO*), homeostatic (*SELL/CXCR2*), and suppressive (*ARG1/CD274/VEGFA*) states (Shen et al., 2025; Xiao et al., 2025a). Rather than fixed lineages, these clusters are better interpreted as infection-stage- and microenvironment-dependent programs.

The 2025 consensus roadmap maps these programs to PMN-I, PMN-im, PMN-IFN, PMN-NET, PMN-b, and PMN-sup labels (Ng et al., 2025). In bacterial pneumonia, immature, ISG-high, and NET-forming states show the clearest association with severe disease, whereas homeostatic states are relatively preserved in milder presentations (Shen et al., 2025; Xiao et al., 2025a).

### 4.2 Association between neutrophil subset proportions and disease severity

The composition of neutrophil subsets is significantly associated with the severity of bacterial pneumonia. The proportion of immature neutrophils in BALF is increased in severely affected patients; these cells are commonly interpreted as emergency granulopoiesis-associated neutrophils and may have reduced functional maturity, but direct phagocytic, migratory, and bactericidal validation in bacterial pneumonia BALF remains limited (Chang et al., 2025; Xiao et al., 2025a). Concurrently, enrichment of ISG-high neutrophils in severely affected patients suggests an interferon-skewed inflammatory program associated with severe disease; whether this program causally impairs bacterial clearance or promotes immune-cell exhaustion requires functional testing (Xiao et al., 2025a). By comparison, the proportion of homeostatic neutrophil programs in BALF from mildly affected patients is relatively higher, suggesting that preservation of balanced neutrophil-state composition may be associated with more effective infection control (Shen et al., 2025). In sepsis-associated pneumonia, single-cell analysis of BALF further identifies neutrophil programs enriched during the immunosuppressive phase; these G-MDSC–like cells may contribute to sepsis-associated immune paralysis, but their suppressive function requires protein-level and functional validation (Shen et al., 2025). This pattern supports cell-subset–based severity assessment and prognostic modeling, but prospective paired-sample validation is still required.

### 4.3 NETs, inflammatory cascades, and tissue injury

NETs are an important effector mechanism by which neutrophils exert their bactericidal function during bacterial infection, but their excessive release also constitutes a key pathological component of lung tissue injury (Shahzad et al., 2025). Single-cell analyses indicate that NET-associated neutrophil programs highly express peptidylarginine deiminase 4 (PADI4); because this enzyme catalyzes citrullination of histone H3 and promotes chromatin decondensation, this transcriptional signature should be paired with citrullinated histone H3 and extracellular DNA/MPO-DNA assays to confirm NET formation. In severe bacterial pneumonia, excessive NETs can directly damage alveolar epithelial and vascular endothelial cells through their network-like DNA scaffolds and the toxic proteins (histones, elastase, MPO) attached to them, and can cause secondary tissue injury by activating the complement system and the coagulation cascade, forming an inflammation–coagulation–injury positive feedback process (Fan et al., 2024; Shahzad et al., 2025). Beyond direct tissue injury, neutrophil bactericidal activity may also be actively suppressed by pathogens. *Staphylococcus aureus* can induce neutrophils to produce itaconate, which suppresses the oxidative burst and represents a pathogen-related immune evasion mechanism: as a tricarboxylic acid (TCA) cycle intermediate that abnormally accumulates, itaconate inhibits succinate dehydrogenase (SDH) activity, blocks the mitochondrial respiratory chain, and weakens neutrophil oxidative bactericidal capacity (Tomlinson et al., 2023). Neutrophil-derived inflammasome assembly also plays an important role in respiratory infections, and the maturation and release of IL-1β and IL-18 mediated by inflammasomes further amplify local inflammation (Hassane et al., 2017). The interplay among these pathways helps explain the clinical observation that neutrophil overactivation in severely affected patients can paradoxically aggravate lung injury: bactericidal effects and tissue injury within the neutrophil functional spectrum are not mutually exclusive but coexist with different intensities across different subsets.

### 4.4 Neutrophils as potential HDT targets

The dissection of neutrophil subset heterogeneity provides new opportunities to refine HDT strategies. Traditional approaches that suppress neutrophils as a whole have lacked subset selectivity and have been limited in balancing efficacy and safety. Single-cell atlas data nominate damage-associated neutrophil programs as candidate intervention nodes, including NET-associated and immunosuppression-associated programs, but selective targeting requires markers and functional assays that can distinguish injurious programs from antibacterial effector functions (Feng et al., 2025; Janciauskiene et al., 2026). In pneumococcal pneumonia models, modulation of specific neutrophil subset phenotypes has been reported to enhance the efficacy of HDT, providing preclinical support rather than direct clinical evidence (Matarazzo et al., 2025). PAD4 inhibitors may reduce NET formation by blocking histone citrullination; CXCR2 antagonists may modulate neutrophil recruitment to the lung; inhaled DNase I may degrade extracellular NET scaffolds and reduce tissue toxicity; and metabolic reprogramming strategies are hypothesized to restore neutrophil oxidative-burst capacity and improve antibacterial performance, but these approaches require direct testing in infection-relevant neutrophil subsets (Chang et al., 2025; Janciauskiene et al., 2026). These strategies have received varying levels of support in preclinical studies. At present, the limiting step is not recognition of NET-high or *ARG1*-high neutrophil programs in scRNA-seq data, but the lack of surface markers, functional validation, and delivery approaches that can separate and manipulate these states in viable cells without compromising bacterial clearance.

## 5 T cells and B cells: exhaustion, immune deficiency, and protective responses

Adaptive immune responses are important for pathogen clearance and immune memory formation in bacterial pneumonia, but single-cell atlas data consistently identify T-cell dysfunction-associated transcriptional signatures in infected lung tissue. In BALF, CD8+ T cells exhibit a marked exhaustion-like transcriptional phenotype, with high expression of multiple inhibitory receptor genes or markers including *PD-1*, *TIM-3*, *LAG-3*, and *TIGIT* (Patera et al., 2016; Xiao et al., 2025a). This exhaustion-like state differs from the classical T-cell exhaustion seen in chronic viral infections, such as human immunodeficiency virus (HIV) and hepatitis C virus (HCV), and tumors: it develops over days rather than weeks to months; its principal drivers may include an inflammatory microenvironment composed of high concentrations of cytokines and immune checkpoint ligands rather than persistent antigen stimulation; and it may be more reversible than chronic exhaustion after infection control, although this reversibility requires longitudinal and functional validation in bacterial pneumonia (Xiao et al., 2025a). Single-cell–level subset analysis further reveals transcriptional heterogeneity within exhausted-like T cells: some cells retain expression of cytotoxic effector genes such as *PRF1* and *GZMB* but show reduced proliferative signatures and are annotated as “progenitor exhausted-like” cells; others display a “terminal exhaustion-like” phenotype with stronger loss of effector-associated programs (Xiao et al., 2025a). Parallel analysis of peripheral blood atlases shows that the proportion of CD38^+^HLA-DR^+^ activated/exhausted T cells is associated with disease severity and prognosis (Patera et al., 2016; Xiao et al., 2025b). This intrasubset heterogeneity supports a more selective framework for testing immune checkpoint modulation: progenitor-exhausted-like cells may represent a more plausible population for checkpoint-response testing, whereas this remains unvalidated in bacterial pneumonia and alternative approaches may be needed for terminally exhausted-like states.

CD4^+^ T cells in bacterial pneumonia also show complex functional differentiation. Th1 cells, a major effector subset of antibacterial immunity, show reduced IFN-γ-related signatures or production in severely affected patients, suggesting possible cell-intrinsic functional impairment that requires protein-level or cytokine-level confirmation (Xiao et al., 2025a). Th17 cells are important for mucosal barrier defense and neutrophil recruitment, but their excessive activation can also exacerbate tissue inflammation (Traber and Mizgerd, 2025). With respect to humoral immunity, follicular helper T cells (Tfh) promote local antibody production through coordinated interactions with B cells in inducible bronchus-associated lymphoid tissue (iBALT). Single-cell ligand–receptor interaction analysis suggests that the CXCL13/CXCR5 and IL-21/IL-21R signaling networks between Tfh cells and plasma cells in BALF of severely affected patients are markedly altered; these changes may be associated with impaired local humoral coordination, but a direct contribution to insufficient antibody responses requires spatial and functional validation (Xiao et al., 2025a). Mucosal-associated invariant T cells (MAITs), an unconventional T cell subset, can rapidly recognize bacterial riboflavin metabolic intermediates through their semi-invariant T cell receptor and exhibit relatively rapid responses in lung infection (Kamiki et al., 2025). Overall, the disrupted coordination among CD4^+^ T cell subsets, and between these subsets and B cells, is an important component of adaptive immune deficiency in severe bacterial pneumonia.

The role of regulatory T cells (Tregs) in infection immunity is not limited to immunosuppression. Single-cell atlases show that the proportion of Treg-associated programs in BALF is significantly increased in severely affected patients, and excessive Treg expansion may suppress effective antibacterial immunity and delay pathogen clearance, although this requires functional confirmation in bacterial pneumonia (Xiao et al., 2025a).

However, Treg function is clearly time-dependent: during the acute phase of infection, Tregs help limit excessive inflammation and protect tissue from immunopathological damage, whereas during the recovery phase Tregs participate in tissue repair (Joudi et al., 2025). The epigenetic basis of this functional transition has been preliminarily elucidated: the reparative function of induced Tregs depends on maintenance DNA methylation, and the loss of this maintenance reduces their capacity to promote tissue regeneration (Joudi et al., 2025). These findings indicate that the protective or pathogenic role of Tregs at different stages of infection depends on the dynamic balance between the inflammatory microenvironment and epigenetic state, rather than on simple changes in cell number.

Based on single-cell evidence regarding exhaustion-like T-cell programs and CD4+ T-cell differentiation, several strategies aimed at testing whether T-cell dysfunction-associated programs can be reversed are being explored. Immune checkpoint blockade, such as anti-PD-1/PD-L1 antibodies, has shown potential to restore selected T-cell readouts in preclinical models of sepsis-associated immune paralysis and in ex vivo studies of patient immune function (Patera et al., 2016; Xiao et al., 2025a), but clinical application requires caution: in a Phase I randomized trial of nivolumab in sepsis, the available data were primarily safety, tolerability, and pharmacodynamic findings, and were insufficient to confirm efficacy; excessive immune activation may aggravate organ injury, and any future use would require immune-state enrichment, low-dose or short-course regimens, and close pathogen-clearance monitoring (Hotchkiss et al., 2019).

As a key cytokine for T-cell survival and proliferation, IL-7 has shown the ability to restore lymphocyte counts in patients with septic shock in the IRIS-7 randomized clinical trial, but its effect on hard endpoints such as mortality, pathogen clearance, secondary infection, and recurrent infection requires confirmation in larger studies (Francois et al., 2018). Targeting epigenetic modifications is another potential intervention pathway: a viral pneumonia mouse model suggests that maintenance DNA methylation is important for the reparative function of induced Tregs (Joudi et al., 2025), but whether DNA methyltransferase (DNMT) inhibitors can safely and selectively reshape exhaustion-like T cells or Treg function in bacterial pneumonia remains to be validated. Given that T-cell dysfunction-associated programs involve multiple inhibitory signals, metabolic abnormalities, and epigenetic regulation, future studies may need to test whether immune checkpoint modulation, cytokine supplementation, or epigenetic intervention can be safely matched to specific immune states without impairing pathogen control.

## 6 Structural cells: injury and repair of epithelial, endothelial, and fibroblast compartments

Lung tissue injury in bacterial pneumonia is not limited to inflammatory cell infiltration and activation. Injury, aberrant state transitions, and impaired repair of three classes of structural cells—epithelial cells, endothelial cells, and fibroblasts—are likely contributors to disease severity and long-term prognosis, although bacterial pneumonia-specific evidence remains less direct than for myeloid-cell programs. Single-cell transcriptomics provides a higher-resolution analytical approach for dissecting the transcriptional heterogeneity and inferred pathological state transitions of structural cells in the infectious microenvironment, and provides a hypothesis-generating evidence base for incorporating structural cells into future HDT validation studies (Whitsett and Alenghat, 2015; Zhang et al., 2025).

### 6.1 Alveolar epithelial injury and impaired regeneration

The integrity of the alveolar epithelial barrier is a prerequisite for gas-exchange function. Bacterial pneumonia can cause death of large numbers of alveolar type 1 cells (AT1) and damage to alveolar type 2 cells (AT2). Under physiological conditions, AT2 cells act as alveolar stem cells and can differentiate into AT1 cells to complete epithelial repair (Whitsett and Alenghat, 2015; Zhang et al., 2025). However, single-cell studies in lung-injury and ARDS-related contexts indicate that the differentiation trajectory of AT2 cells can become markedly aberrant: a subset of AT2 cells may be arrested in an aberrant transitional state, highly expressing basal-cell or transitional markers such as *KRT8* and *TP63* but failing to complete terminal differentiation into AT1 cells, which is interpreted as a repair-failed or delayed-regeneration program (Chen et al., 2024; Zhang et al., 2025). This arrest may be related to suppression of pro-differentiation pathways such as Wnt/β-catenin in severe infection, suggesting that Wnt pathway modulation is a candidate repair hypothesis derived mainly from lung-regeneration biology; however, bacterial pneumonia-specific efficacy and safety remain untested (Zhang et al., 2025).

Lung epithelial cells are not passive injury targets but active participants in the immune response. Complement C3 produced by epithelial cells has been reported to enhance antibacterial defense in the alveolar space, and epithelium-derived *Sectm1a* participates in regulating neutrophil recruitment to the infection site (Sahu et al., 2023; Tanaka et al., 2025). With regard to macrophage–epithelial interactions, macrophage-derived oncostatin M (OSM) has been shown in lung-injury contexts to support lung epithelial barrier repair, indicating that macrophage support of epithelial regeneration may be an important component of barrier repair (Hoagland et al., 2025). At the airway level, club cells secrete protective proteins such as CC16 to maintain airway surface liquid homeostasis, whereas excessive proliferation of goblet cells (goblet cell metaplasia) can lead to mucus hypersecretion and airway obstruction; scRNA-seq studies have identified the aberrant state transitions of these cells during infection (Shan et al., 2024).

### 6.2 Endothelial barrier dysfunction and increased vascular permeability

Lung endothelial cells participate in maintaining vascular barrier integrity and regulating transendothelial migration of immune cells (Vila Ellis et al., 2025). In bacterial pneumonia and sepsis, endothelial barrier dysfunction is a key pathological component leading to pulmonary edema and ARDS. Recent studies have implicated endothelial C-type lectin domain family 5 member A (CLEC5A) as a candidate mediator of barrier dysfunction and increased vascular permeability: activation of CLEC5A has been associated with degradation of inter-endothelial junction proteins, such as VE-cadherin, and cytoskeletal rearrangement, thereby increasing vascular permeability (Zhang et al., 2025). Single-cell analysis further suggests transcriptional heterogeneity among endothelial cells in infected lung tissue: one endothelial program upregulates adhesion molecules such as ICAM-1 and VCAM-1 and may promote immune-cell recruitment, whereas another program shows barrier-dysfunction-associated and procoagulant signatures, which may be associated with microthrombus formation and the risk of disseminated intravascular coagulation (DIC) (Vila Ellis et al., 2025). Given the important pathological role of endothelial barrier dysfunction in severe pneumonia, CLEC5A inhibition represents a candidate barrier-protective strategy supported by animal models of ARDS or acute lung injury, but its therapeutic relevance in bacterial pneumonia requires direct validation (Zhang et al., 2025).

### 6.3 Fibroblast heterogeneity and repair failure

Fibroblasts have long been viewed as passive structural support cells in the lung, but single-cell atlas studies have substantially altered this view. Cross-tissue fibroblast atlases and lung single-cell data reveal pronounced heterogeneity among lung fibroblasts: lipofibroblasts participate in lipid metabolism and surfactant homeostasis; myofibroblasts contribute to extracellular matrix (ECM) contraction and remodeling; and inflammatory fibroblasts participate in immune cell recruitment by secreting chemokines (Ghonim et al., 2023; Liu et al., 2025). During the acute phase of infection, fibroblasts secrete ECM components that support tissue repair and regulate epithelial regeneration through platelet-derived growth factor (PDGF) and Wnt signaling (Chen et al., 2024).

When infection persists or inflammatory responses are excessive, fibroblasts can differentiate toward an aberrantly activated, profibrotic phenotype. Such cells overproduce collagen (*COL1A1*, *COL3A1*) and tissue inhibitors of metalloproteinases (TIMPs), suppressing normal ECM degradation and remodeling and ultimately leading to lung fibrosis and repair failure (Ghonim et al., 2023; Jiang et al., 2025). The transition from physiological repair to pathological fibrosis represents a candidate lower-tier treatable cell state at the structural-cell level: TGF-β pathway modulation, antifibrotic agents such as pirfenidone and nintedanib, and anti-PDGF strategies have accumulated pharmacological evidence for suppressing fibroblast profibrotic differentiation in fibrotic lung disease or related contexts, but bacterial pneumonia-specific validation is still required (Ghonim et al., 2023; Jiang et al., 2025).

### 6.4 Cross-system interactions between structural and immune cells and neuroimmune regulation

Injury and repair of structural cells do not occur in isolation but are functionally coupled with immune cells. In addition to macrophage-derived OSM-associated epithelial barrier repair, recent studies of the brain–lung axis add a neuroimmune dimension to structural cell–immune cell interactions: GABAergic neurons can regulate the intensity of pulmonary inflammatory responses through ADRB2+ interstitial macrophages, suggesting that the nervous system may remotely modulate the lung tissue repair microenvironment (Li et al., 2025). For translation, these structural programs should be treated as validation candidates: the immediate question is which markers can be monitored during acute infection and which repair pathways can be modulated without aggravating fibrosis or impairing host defense (Jiang et al., 2025; Zhang et al., 2025; Zhang et al., 2025).

## 7 Cell–cell interaction network and cytokine cascade

Ligand–receptor co-expression analyses based on single-cell transcriptomic data predict that immune cells and structural cells in BALF of bacterial pneumonia may form a multilayered communication network, comprising several candidate interaction modules: immune checkpoint interactions between macrophages and T cells (PD-L1/PD-1; Galectin-9/TIM-3); repair-associated signaling between macrophages and epithelial cells (OSM/OSMR); transendothelial migration-associated signaling between neutrophils and endothelial cells (ICAM-1/LFA-1); and regenerative signaling between fibroblasts and epithelial cells (hepatocyte growth factor [HGF]/c-Met; Wnt/Frizzled) (Shan et al., 2024; Hoagland et al., 2025; Xiao et al., 2025a). In severely affected patients, the inferred topology of this interaction network appears to be markedly remodeled: proinflammatory and immunosuppression-associated signaling pathways are simultaneously enhanced, whereas tissue-repair and homeostatic-maintenance signatures are correspondingly attenuated, reflecting an overall imbalance of immune homeostasis. However, computational strategies that infer protein interactions from transcriptomic levels have inherent limitations: mRNA expression and protein abundance are not always linearly correlated; functionally important but lowly expressed ligand–receptor pairs may be missed; and scRNA-seq lacks spatial coordinate information and cannot directly verify whether interacting cells are within physical contact distance in tissue (Lyu et al., 2025). Therefore, key interaction pairs identified by computational analysis still require validation by spatial transcriptomics, fluorescence resonance energy transfer, or *in situ* hybridization to distinguish genuine intercellular signaling events from coincidental transcriptional co-expression.

Within this interaction landscape, severe disease is marked by coupled amplification of inhibitory immune signaling and attenuation of repair-associated signaling, together with rapid cytokine release. Single-cell atlases infer likely cytokine-producing programs: *IL1B* and *TNF* transcripts are enriched in inflammatory macrophage/neutrophil states, *IL6* in monocyte-derived macrophages and activated fibroblasts, and *IL10* in Tregs and selected macrophage states (Xiao et al., 2025a; Fan et al., 2024). In parallel, *S100A8/A9*-high blood monocytes support spillover into systemic inflammation through the TLR4–MYD88 axis (Xiao et al., 2025b). The practical use of this mapping is to define testable source-target pairs for intervention and monitoring, not to justify non-selective immunosuppression.

For bacterial pneumonia, the practical value of ligand–receptor analysis is not to prove cell communication by itself, but to nominate interaction pairs for spatial or functional validation. At present, PD-L1/PD-1, OSM/OSMR, ICAM-1/LFA-1, and CLEC5A-linked endothelial injury are stronger validation priorities than immediate therapeutic targets. Their translational value will depend on paired BALF–blood or tissue-level validation and on whether shifts in these pairs track treatment response rather than disease severity alone. Thus, ligand–receptor pairs and cytokine-source assignments should be treated as testable hypotheses rather than evidence of functional communication by themselves.

## 8 Prioritizing treatable cell states for HDT strategies

We use “treatable cell states” as a translational prioritization scheme rather than a binary label of clinical readiness. To make the evidence-ranking system explicit, candidate cell states were assigned to tiers according to six parameters: (i) directness of human evidence, with human BALF or lung tissue data weighted more strongly than peripheral blood, animal-only evidence, or adjacent-disease evidence; (ii) cohort scale and reproducibility across independent datasets; (iii) disease-context specificity, including whether the evidence was derived from bacterial pneumonia itself or extrapolated from sepsis, ARDS, viral pneumonia, tuberculosis, tumor immunology, or fibrotic lung disease; (iv) availability of clinical modifiers, including age, pathogen type, coinfection, disease severity, and underlying conditions where reported; (v) validation beyond transcript abundance, including surface protein expression, secreted mediators, metabolite readouts, spatial localization, or functional assays; and (vi) availability of plausible intervention evidence and safety data. Because the included studies do not uniformly report age structure, coinfection, comorbidities, pathogen burden, or paired BALF–blood data, the tier system should be interpreted as a transparent evidence-prioritization framework rather than as a formal clinical recommendation grade.

Under this framework, Tier 1 represents candidate states supported by direct human bacterial pneumonia atlas evidence, severity association, reproducible markers, and convergent mechanistic or preclinical evidence. Tier 2 represents states with human transcriptomic support and plausible intervention evidence, but incomplete protein-level, functional, or clinical validation. Tier 3 represents states for which bacterial pneumonia-specific evidence remains limited and is partly extrapolated from adjacent lung-injury contexts. Accordingly, IDO1+/PD-L1+ macrophages and pathological neutrophils are classified as Tier 1; M-MDSC-like macrophage states and exhausted-like T-cell states are classified as Tier 2; and injured epithelial and repair-failed stromal states are classified as Tier 3. The key task is therefore not to enumerate additional states, but to test reversibility, functional relevance, and intervention responsiveness in paired longitudinal cohorts.

**Figure 3:**
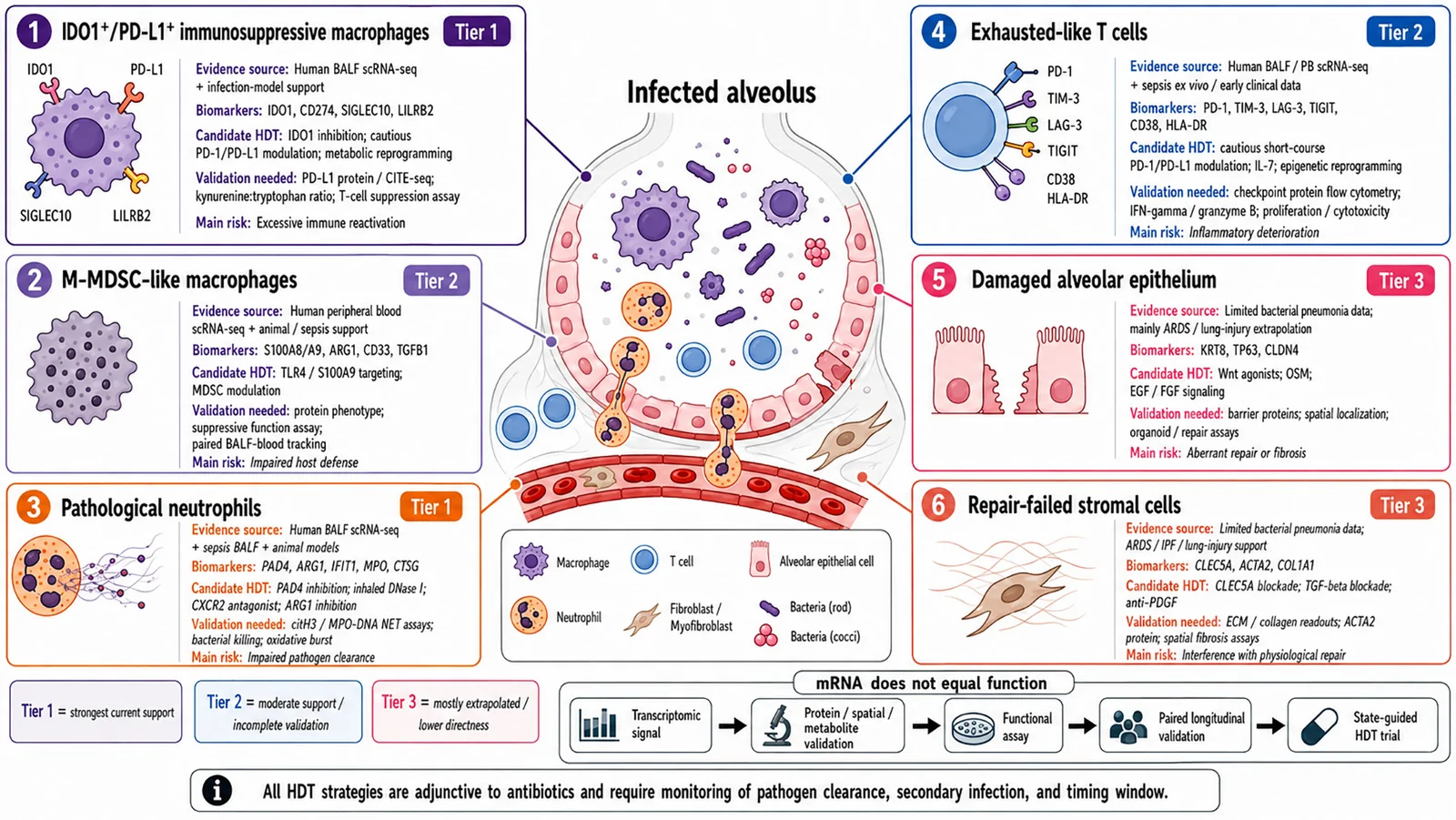
Evidence-tiered candidate treatable cell states and validation requirements for host-directed therapy in bacterial pneumonia. The figure summarizes six candidate cell states across macrophage, neutrophil, T-cell, epithelial, endothelial, and stromal compartments. Tier assignment reflects directness of human evidence, cohort scale, disease-context specificity, availability of clinical modifiers, validation beyond transcriptomics, intervention plausibility, and safety considerations. Tier 1 states have the strongest current support from human bacterial pneumonia single-cell datasets and convergent mechanistic evidence; Tier 2 states have human transcriptomic support but incomplete protein-level, functional, or clinical validation; Tier 3 states remain partly extrapolated from adjacent lung-injury contexts. Candidate HDT options should therefore be interpreted as hypotheses requiring protein-level, spatial, functional, and longitudinal validation, not as established treatment recommendations. Potential risks include impaired pathogen clearance, secondary infection, excessive immune activation, and time-dependent therapeutic effects.

Using this prioritization logic, we organize six candidate treatable cell states across innate immune, adaptive immune, and structural compartments. **Figure 3** and **Table 1** were revised to present these states as evidence-tiered candidates rather than validated therapeutic targets. **Figure 3** highlights the evidence source, validation requirements, and major safety concerns for each candidate state, whereas **Table 1** provides a structured summary of markers, intervention hypotheses, validation status, and evidence tier.

**Table 1:** Evidence-tiered candidate treatable cell states and host-directed therapy strategies in bacterial pneumonia.

Treatable cell state	Molecular markers	Inferred pathological mechanism	Primary evidence source	Validation beyond transcriptomics	Candidate HDT strategies	Main safety concern	Evidence tier/developmental stage	Key references
IDO1+/PD-L1+ immunosuppressive macrophages	*IDO1, CD274, SIGLEC10, LILRB2*	Tryptophan depletion and PD-L1-mediated T-cell suppression; proposed IDO1–kynurenine–AhR and PD-1/PD-L1 inhibitory signaling	Human BALF scRNA-seq in bacterial pneumonia; supported by infection-model evidence	Needs IDO1 protein detection, PD-L1 surface expression by flow cytometry or CITE-seq, kynurenine/tryptophan ratio, macrophage–T-cell suppression assay, and phagocytosis assay	IDO1 inhibitors; cautious low-dose anti-PD-1/PD-L1 modulation; metabolic reprogramming	Excessive immune reactivation, worsening inflammatory lung injury, and uncertain timing relative to pathogen burden	Tier 1; preclinical/Phase I	(Xiao et al., 2025a; Xu et al., 2025)
M-MDSC-like macrophages	*S100A8/A9*, ARG1, CD33, TGFB1	*S100A8/A9*–TLR4–MYD88-driven systemic inflammation and immunosuppressive myeloid expansion; ARG1-associated arginine depletion may suppress T-cell responses	Human peripheral blood scRNA-seq; animal and sepsis-related support; limited direct BALF validation in bacterial pneumonia	Needs protein-level MDSC phenotype, ARG1 protein/activity, suppressive function assay, paired BALF–blood tracking, and pathogen-clearance correlation	TLR4 antagonists; anti-S100A9 neutralizing antibodies; MDSC modulation or depletion strategies	Impaired host defense, delayed bacterial clearance, and increased risk of secondary infection	Tier 2; preclinical	(Feng et al., 2025; Xiao et al., 2025b; Xu et al., 2025)
Pathological neutrophils	*PADI4, ARG1, IFIT1, MPO, CTSG*	NET overproduction, immature or ISG-high neutrophil expansion, tissue injury, and possible immunosuppressive neutrophil programs	Human BALF scRNA-seq in bacterial pneumonia; sepsis-associated BALF studies; animal pneumonia models	Needs citrullinated histone H3, MPO–DNA complexes, extracellular DNA quantification, oxidative burst assay, bacterial killing assay, and epithelial/endothelial injury assay	PAD4 inhibitors; inhaled DNase I; CXCR2 antagonists; ARG1 inhibitors	Impaired pathogen clearance if neutrophil recruitment, NET formation, or bactericidal function is over-suppressed	Tier 1; preclinical/Phase I	(Matarazzo et al., 2025; Shahzad et al., 2025; Shen et al., 2025; Xiao et al., 2025a)
Exhausted-like T cells	PD-1, TIM-3, LAG-3, TIGIT, CD38, HLA-DR	Multiple inhibitory receptor expression associated with reduced cytotoxicity, impaired proliferation, and immune-paralysis-like adaptive dysfunction	Human BALF and peripheral blood scRNA-seq; sepsis ex vivo studies; early clinical immune-restoration studies	Needs checkpoint protein flow cytometry, IFN-gamma production, granzyme B/perforin expression, proliferation assay, cytotoxicity assay, and response to checkpoint or IL-7 stimulation	Low-dose short-course anti-PD-1/PD-L1 modulation; IL-7 supplementation; epigenetic reprogramming	Inflammatory deterioration, excessive immune activation, and organ injury if used during uncontrolled hyperinflammation	Tier 2; Phase I/Phase II	(Patera et al., 2016; Francois et al., 2018; Hotchkiss et al., 2019; Antcliffe et al., 2025; Joudi et al., 2025; Xiao et al., 2025a; Xiao et al., 2025b)
Injured alveolar epithelium	*KRT8, TP63, CLDN4*	AT2 transitional-state arrest, failed AT2-to-AT1 differentiation, epithelial barrier dysfunction, and impaired alveolar regeneration	Limited bacterial pneumonia-specific evidence; mainly extrapolated from ARDS and lung-injury datasets	Needs epithelial protein-marker validation, spatial localization, barrier integrity assays, organoid or ex vivo repair assays, and longitudinal association with oxygenation or resolution	Wnt agonists; oncostatin M; EGF/FGF signaling modulation	Aberrant repair, mucus or epithelial remodeling, and potential promotion of fibrosis if repair signaling is mistimed or excessive	Tier 3; preclinical	(Chen et al., 2024; Hoagland et al., 2025; Zhang et al., 2025)
Repair-failed stromal cells	*CLEC5A, ACTA2, COL1A1*	Barrier dysfunction, endothelial injury, myofibroblast activation, excessive extracellular-matrix deposition, and profibrotic repair failure	Limited bacterial pneumonia-specific evidence; mainly extrapolated from ARDS, IPF, sepsis-associated lung injury, and lung-fibrosis studies	Needs CLEC5A protein validation, VE-cadherin or endothelial-barrier readouts, ACTA2 protein, collagen deposition, spatial fibrosis assays, and ECM-remodeling assessment	CLEC5A inhibitors; TGF-beta blockers; anti-PDGF agents	Interference with physiological repair, impaired barrier restoration, and uncertainty in timing of anti-fibrotic intervention	Tier 3; preclinical/marketed in other indications	(Ghonim et al., 2023; Jiang et al., 2025; Zhang et al., 2025)

Evidence tier definitions: Tier 1 = strongest current support, including direct human bacterial pneumonia single-cell evidence, severity association, reproducible markers, and convergent mechanistic or preclinical support. Tier 2 = moderate support, including human transcriptomic evidence but incomplete protein-level, functional, or clinical validation. Tier 3 = lower directness, where bacterial pneumonia-specific evidence remains limited and is partly extrapolated from adjacent lung-injury contexts.

HDT, host-directed therapy; BALF, bronchoalveolar lavage fluid; PB, peripheral blood; NET, neutrophil extracellular trap; ARDS, acute respiratory distress syndrome; IPF, idiopathic pulmonary fibrosis; ECM, extracellular matrix. Candidate HDT strategies should be interpreted as hypothesis-generating and adjunctive to antimicrobial therapy, not as validated treatment recommendations.

For IDO1+/PD-L1+ macrophages, IDO1 inhibition and checkpoint modulation are mechanistically plausible, but negative oncology experience with epacadostat cautions against assuming target engagement equals clinical benefit; in infection, the key safety risk is excessive immune reactivation (Xiao et al., 2025a; Xu et al., 2025). For M-MDSC-like macrophages, modulation of the S100A8/TLR4 axis is supported preclinically, but broad TLR4 blockade may impair host defense, delay bacterial clearance, or increase susceptibility to secondary infection (Feng et al., 2025; Xiao et al., 2025b; Xu et al., 2025). For pathological neutrophils, PAD4/NET and ARG1/CXCR2 directions are plausible, but the central tradeoff is whether tissue injury can be reduced while preserving bacterial clearance, oxidative burst, and early pathogen containment (Chang et al., 2025; Matarazzo et al., 2025; Shahzad et al., 2025).For exhausted-like T cells, IL-7 and cautious low-dose checkpoint modulation show early translational signals, but effects on hard clinical outcomes, pathogen clearance, secondary infection, recurrence, and organ injury remain uncertain (Patera et al., 2016; Francois et al., 2018; Hotchkiss et al., 2019; Antcliffe et al., 2025; Joudi et al., 2025). For injured epithelium and repair-failed stromal states, pro-repair and anti-fibrotic options are biologically plausible, yet evidence in bacterial pneumonia remains partly extrapolated and requires disease-specific validation, especially to avoid mistimed repair stimulation, impaired physiological repair, or fibrosis-promoting effects (Chen et al., 2024; Jiang et al., 2025; Zhang et al., 2025; Zhang et al., 2025).

Risk–benefit balance is central to any HDT strategy in bacterial pneumonia. Suppressing inflammatory pathways may reduce epithelial or endothelial injury, but may also impair bacterial clearance, prolong pathogen persistence, or increase susceptibility to secondary and recurrent infections. Conversely, immune-restorative strategies such as checkpoint modulation or cytokine supplementation may improve antimicrobial immunity in selected immunosuppressed phenotypes, but may aggravate inflammatory lung injury if administered during an uncontrolled hyperinflammatory phase. Therefore, HDT should be considered only as an adjunct to effective antimicrobial therapy and should be evaluated with pathogen-load monitoring, microbiological clearance, secondary infection rates, recurrence, organ injury markers, and longitudinal immune-state readouts. The therapeutic window is likely narrow and state-dependent: early high-burden infection may require preservation of innate clearance, whereas later immune-paralysis phenotypes may be more suitable for carefully titrated immune restoration.

These evidence and safety boundaries are integrated into the revised **Figure 3**, which presents candidate states as evidence-tiered hypotheses requiring transcriptomic, protein-level, spatial, functional, and longitudinal validation before state-guided HDT testing.

## 9 Clinical challenges and future directions

Clinical translation now depends on three bottlenecks rather than additional cataloging. First is the time bottleneck: full scRNA-seq workflows require days to weeks, whereas severe bacterial pneumonia often demands treatment decisions within hours. Second is the sampling bottleneck: BALF provides lung-local resolution but is invasive, and whether peripheral blood signatures can reliably represent lung states remains unproven without paired BALF–blood cohorts. Third is the causal bottleneck: most associations between cell states and severity remain observational, so targetability and safety are still uncertain.

Accordingly, the near-term goal should be pragmatic rather than comprehensive: build fast assays for a small state panel and validate them prospectively against outcomes. Candidate implementations include rapid multiparameter flow cytometry and targeted host-response panels that can return results within a clinically relevant window, while using paired longitudinal sampling to anchor assay outputs to lung-local biology.

For intervention studies, the focus should shift from discovering more subsets to testing whether state-guided stratification changes treatment effects. A workable first pass is classification into immunosuppressive, hyperinflammatory, and repair-impaired phenotypes, followed by matched HDT hypotheses and prespecified safety monitoring. This strategy remains provisional and should be interpreted as a trial design scaffold rather than a ready-to-implement clinical protocol.

**Figure 4:**
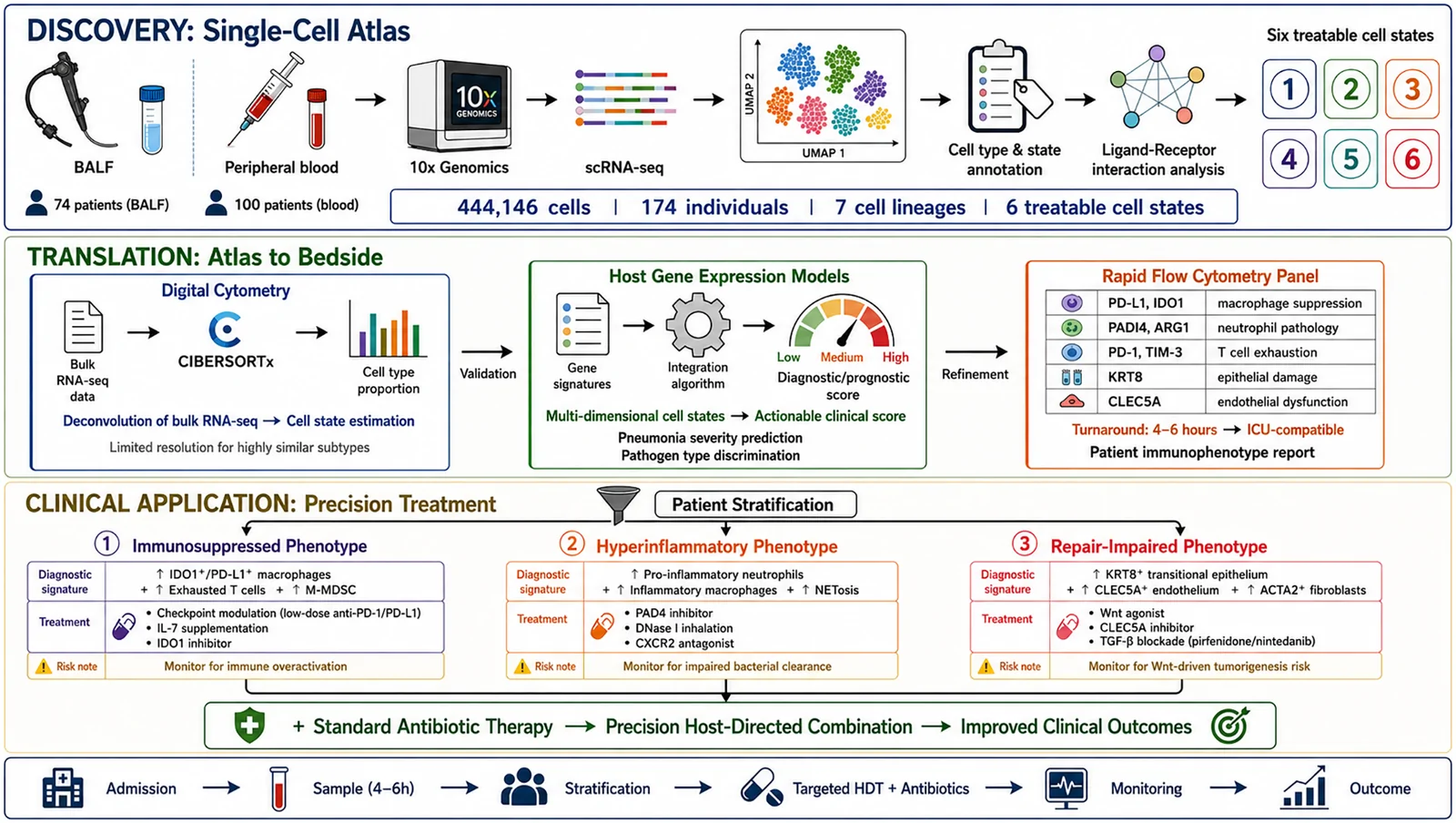
Proposed route from single-cell atlases to state-guided HDT in bacterial pneumonia. Atlas-derived immune and structural cell states are converted into candidate biomarkers using digital cytometry, host-response models, or rapid flow cytometry, followed by provisional stratification into immunosuppressive, hyperinflammatory, and repair-impaired phenotypes. The scheme is hypothetical and requires validation in paired BALF–blood cohorts and prospective intervention studies.

In this context, **Figure 4** presents a tentative route from atlas-derived states to bedside stratification, with explicit dependence on paired BALF–blood validation and prospective interventional studies before routine ICU deployment (**Figure 4**).

## 10 Conclusion

Single-cell atlases have shifted the main question in bacterial pneumonia from how many inflammatory cells are present to which transcriptionally defined pathological cell programs dominate the infected lung and whether they correspond to validated functional states. The most reproducible signals to date are immunosuppressive macrophage and pathological neutrophil programs, with complementary evidence for exhausted-like T-cell states and structurally injured epithelial/stromal compartments.

In this review, “treatable cell states” is used as a target-prioritization scheme rather than as a mature clinical instrument. Its value is to rank cell programs by evidence strength, translational readiness, validation depth, and safety uncertainty, while keeping weakly supported or extrapolated links explicit.

The next decisive tests are practical and safety-oriented: whether these states can be measured quickly, validated at protein and functional levels, tracked longitudinally, and safely manipulated without compromising pathogen clearance when combined with antimicrobial therapy.
